# The Role of Chalcogen in the ROS Scavenging Mechanism of Model Phenyl Compounds

**DOI:** 10.3390/molecules30071408

**Published:** 2025-03-21

**Authors:** Davide Zeppilli, Veronica Pedergnana, Matteo Filippi, Laura Orian

**Affiliations:** Dipartimento di Scienze Chimiche, Università degli Studi di Padova, Via Marzolo 1, 35131 Padova, Italy; davide.zeppilli@studenti.unipd.it (D.Z.); veronica.pedergnana@studenti.unipd.it (V.P.); matteo.filippi.1@studenti.unipd.it (M.F.)

**Keywords:** activation strain analysis, antioxidant, chalcogen, hydrogen atom transfer, proton electron transfer, ROS scavenging

## Abstract

Phenolic compounds are important antioxidants with great ROS scavenging potential and the presence of the hydroxyl groups is fundamental for this chemical activity. Therefore, changing the chalcogen atom (oxygen) with any of its siblings of group 16 (sulfur, selenium and tellurium) may affect the reactivity of these compounds. In this work, the ROS scavenging activity and mechanism of phenyl chalcogenols was evaluated in silico, unravelling better performance with heavier chalcogens, both thermodynamically and kinetically. Furthermore, a scavenging mechanism switch is reported, moving from Concerted Proton Electron Transfer (CPET) in phenols to Hydrogen Atom Transfer (HAT) in the other phenyl chalcogenols. Both kinetic trends and mechanistic features are rationalized in the framework of Activation Strain Analysis (ASA). Lastly, the role of aromaticity is evidenced by analyzing the differences between the phenol/phenoxyl and methanol/methoxyl self-exchange reactions, as well as between the corresponding processes with the other chalcogens.

## 1. Introduction

A large number of reactions occur simultaneously in the cellular environment. In order to maintain homeostasis, a redox equilibrium is required. Among the various species which are produced, free radicals and peroxides are of interest. An excess of these highly reactive species brings the cells to an oxidative stress condition which represents a risk for the organism [1,2]. Oxidative stress has been observed in neurodegenerative diseases such as Parkinson’s or Alzheimer’s diseases, as well in mood disorders, rheumatoid arthritis, cardiovascular diseases, diabetes and in some cancers [3,4,5,6,7,8,9,10,11]. However, it is not clear if oxidative stress is the cause or merely a symptom of the pathology [1].

From a chemical point of view, some free radicals may be highly reactive species due to the presence of unpaired electrons. Among these types of free radicals, Reactive Oxygen Species (ROSs) are ubiquitous due to the physiological presence of oxygen in aerobic metabolism, which necessarily involves the production of ROSs [12]. The high electronegativity of oxygen makes the ROSs strong electrophiles. For instance, the hydroxyl radical (OH^•^) has the highest electrophilic character [13] and it damages many macromolecular structures through reactions close to the diffusion-limited regime [14]. Conversely, peroxyl radicals are less reactive and stable enough to spread in physiological environments [15,16].

Cells have developed different strategies to defend themselves against these reactive species [17]. Particularly, endogenous macromolecules like enzymes (e.g., superoxide dismutase SOD and glutathione peroxidases GPx) [18,19,20,21] and non-enzymatic species (e.g., glutathione and melatonin) perform antioxidant actions [22,23,24,25]. Moreover, exogenous antioxidants (e.g., some vitamins, carotenoids and polyphenols) are found in food and they may act as radical scavengers by quenching free radicals [26]. However, in physiological conditions, a low steady-state concentration of these reactive species can be essential; for instance, H_2_O_2_ is exploited by cells for redox signaling [27]. Thus, the concentration of peroxide species and free radicals makes the difference between properly working cells and those in oxidative stress conditions for the entire organism. Hence, oxidative stress is an important research field for the development of new drugs.

Among the exogenous radical scavengers, polyphenolic antioxidants are widely studied due to their abundance in nature. Phenolic acids, flavonoids, tannins, coumarins, lignans, quinones, stilbenes and curcuminoids are commonly found in plants [28] and consumed by mammals in fruits and vegetables [29]. Polyphenols are undoubtedly recognized as efficient antioxidant species suppressing or delaying autoxidation of biomolecules through different reaction mechanisms [30]. However, they also exhibit a possible prooxidant activity in presence of transition metals by oxidizing them [31]. Indeed, if the radical product is not stable enough, it may further react with molecular oxygen, propagating the formation and diffusion of free radicals.

**Scheme 1:**
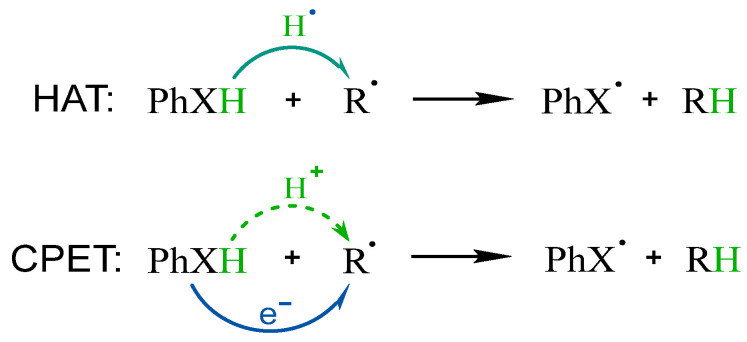
HAT and CPET mechanisms for a phenyl chalcogenol PhXH (X = O, S, Se, Te) and free radical R^•^.

Several possible mechanisms may be involved in the ROS scavenging reactions. In general, polyphenols can be deprotonated in polar environment at physiological pH; then, they may undergo electron transfer, like in Sequential Proton Loss Electron Transfer (SPLET) [14,32,33]. However, for neutral phenolic-based molecules, the most relevant mechanisms are Hydrogen Atom Transfer (HAT) and Concerted Proton Electron Transfer (CPET). They both are single step mechanisms in which a proton and an electron are transferred from a donor to an acceptor; thus, they are thermodynamically indistinguishable (Scheme 1). More precisely, HAT consists in the transfer of a hydrogen atom, i.e., proton and electron are transferred together. Conversely, in CPET, the two entities are transferred separately, usually involving different molecular moieties in the two processes [34,35,36]. Therefore, the distinction between these two mechanisms implies the analysis of the electron flow along the reaction path.

In this work, we focused on the in silico ROS scavenging activity and mechanism of the minimal chemical scaffold of polyphenols, that is the simple phenol. Furthermore, the other chalcogens (S, Se and Te) were included by replacing oxygen to evaluate the effect on the reactivity. Indeed, sulfur and selenium were selected by nature in endogenous antioxidant defenses, like glutathione and the GPx family [18,20,37,38,39,40]. These chalcogens have attracted the attention of the scientific community for the preparation of low-molecular-weight compounds able to mimic the activity of GPx or other enzymes [41,42,43,44,45]. Besides these applications, the presence of a different chalcogen may influence the ROS scavenging activity of simple molecules, as already reported for selenium [46,47,48]. Overall, the strategy of substituting one chalcogen with another one is commonly done in the literature for many molecular classes (phenothiazine [49], quercetin [50], resveratrol [51]), managing to improve their chemical and biological activity. Computational methods are powerful tools to test the chalcogen effect on chemical reactivity, as a screening for a possible in vitro or in vivo application.

This manuscript is organized as follows: The mechanism of scavenging the hydroperoxide radical by the four phenyl chalcogenols is investigated through an accurate description of the energetics and rationalized using an IBO (Intrinsic Bond Orbital)/ASA (Activation Strain Analysis)/EDA (Energy Decomposition Analysis) approach [52]. The role of the aromatic moiety is then elucidated by analyzing the phenol/phenoxyl self-exchange reaction and the methanol/methoxyl self-exchange reaction and the analogous reactions with the heavier chalcogens.

## 2. Results

### 2.1. ROS Scavenging Activity and Mechanism of PhXH (X = O, S, Se, Te)

**Scheme 2:**
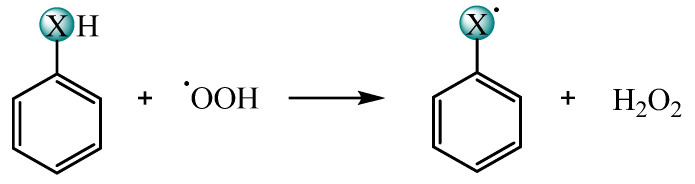
Hydrogen abstraction from phenyl chalcogenols PhXH (X = O, S, Se and Te) by ^•^OOH.

The phenolic moiety represents the chemical scaffold of polyphenols and many other ROS scavengers [29,53,54,55,56,57,58,59]. In fact, the ability of these molecules to quench free radicals relies on the combination of an aromatic moiety and hydroxyl groups. Here, we investigated the role of the chalcogen by replacing the oxygen atom with its heavier siblings of group 16, i.e., sulfur, selenium and tellurium. Particularly, to evaluate the scavenging potential of these model phenyl chalcogenols, the ^•^OOH radical was chosen for the hydrogen abstraction (Scheme 2), since hydroperoxyl radicals are less reactive compared to other ROSs and only very efficient scavengers are able to quench them [15].

First, the thermodynamic feasibility of these reactions was assessed. Table 1 shows the reaction energies of each chalcogenol; both electronic and Gibbs free energies are reported. According to electronic reaction energies in the gas phase, the process becomes more energetically favored with heavier chalcogens. Particularly, the reaction is disfavored (4.5 kcal mol^−1^) only with phenol, while ΔE becomes negative with the other chalcogens, reaching −29.1 kcal mol^−1^ with the tellurol. Moreover, benzene and especially water solvation has a significant effect in favoring the reaction with phenol, which becomes almost isoenergetic in the latter medium (0.6 kcal mol^−1^). Conversely, solvation effects are negligible when oxygen is replaced by any other chalcogen; in general, the reaction energies are slightly more negative.

When considering Gibbs free energies, no significant difference was found. Since the molecularity does not change during the process, the entropy effects are not remarkable and, as a general trend, the reactions become less thermodynamically favored, especially with heavier chalcogens. However, the hydrogen abstraction from phenol is still endergonic and becomes more and more exergonic when going from S to Se and Te, regardless of the environment. Therefore, the phenol is not able to efficiently quench hydroperoxyl radicals, but this process becomes feasible by increasing the number of hydroxyl groups, i.e., in polyphenols [29,53,54] or, as shown here, by replacing oxygen with heavier chalcogens. This observed trend cannot be explained by the increased acidity of heavier chalcogens since the process is not simply a proton transfer. Indeed, the hydrogen abstraction also involves electron transfer; therefore, the X–H bond strength decreases along the group 16. This result is consistent with the analysis of the Bond Dissociation Enthalpies (BDEs) of phenyl chalcogenols, showing lower values in the presence of heavier chalcogens [60].

To complete this picture, the kinetics of these processes can be discussed referring to the transition state energies reported in Table 2. Like the thermodynamic data, these energy values show a straightforward trend: the energy barriers become smaller when moving down along the chalcogen group, regardless of the solvent. Particularly, the electronic energies in the gas phase decrease from 10.8 kcal mol^−1^ (phenol) to 1.4 kcal mol^−1^ (tellurol), while more positive values of 2–3 kcal mol^−1^ are computed in the condensed phase. However, these values should be taken cautiously as effective energy barriers since a stabilized reactant complex may form thanks to the interaction between the two reagents. Therefore, Gibbs free activation energies are more insightful, since the reactant complexes (as well as the transition states) are destabilized by entropic effects and free reactants represent the most stable initial state. Importantly, no substantial difference in the energy trend was observed for Gibbs free activation energies; the highest barrier was computed for phenol (18.6 kcal mol^−1^) and the smallest one is associated with the hydrogen abstraction form tellurol (11.8 kcal mol^−1^). Moreover, the inclusion of water solvation slightly increases all activation energies, as well as the benzene solvation, even though the effect is less pronounced in the latter medium.

**Figure 1:**
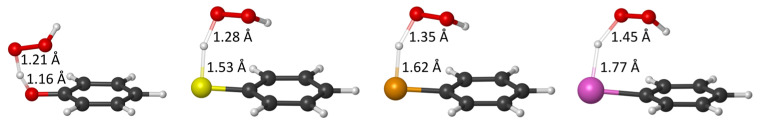
Fully optimized structure of the transition states for the hydrogen abstraction from phenyl chalcogenol by ^•^OOH. X–H distances are reported in Å. Level of theory: M06-2X/6-31G(d),cc-PVTZ-pp.

The fully optimized structures of each transition state are reported in Figure 1. All structures are characterized by an X–H elongation with respect to the corresponding distance in the reactants. Particularly, the X–H distance increases when moving form lighter chalcogens to heavier ones, due to the increased size of the involved atom. Similarly, the distance between the phenyl chalcogen and the ^•^OOH radical (i.e., O–H distance) also increases when heavier chalcogens are present. This indicates that the hydrogen abstraction occurs at the lowest degree of O–H formation for Te, which may be considered more reactant-like. However, this does not explain the energy trend, whose rationalization needs more sophisticated analysis, as reported in the next section.

**Figure 2:**
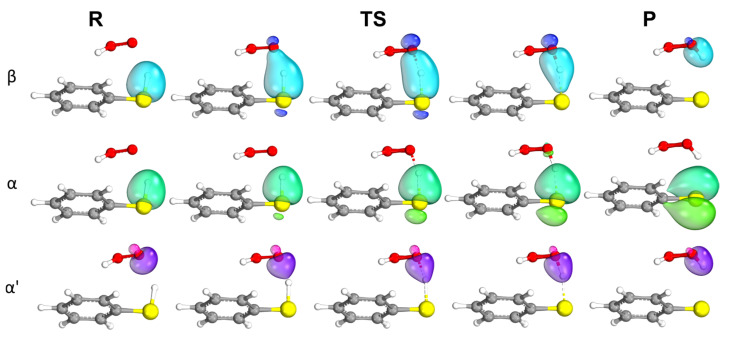
Changes in the main spin IBOs involved in the hydrogen abstraction from phenyl thiol by ^•^OOH along the reaction path: β spin IBO (blue) transferred from the S–H σ bond to the peroxyl radical, the corresponding α spin IBO (green) and the O-centered α′ spin IBO (purple) of the peroxyl moiety. Analogous IBOs are found for Se and Te compounds. Level of theory: M06-2X/def2TZVP//M06-2X/6-31G(d).

The quenching of ^•^OOH by phenyl chalcogenols is referred to as hydrogen abstraction so far since no description of how the electron and the proton are transferred has been presented. To gain insight into this mechanistic aspect, the electron flow along the reaction path must be observed to understand if these processes are described by HAT or CPET mechanism. To this purpose, the main spin IBOs involved in the reactivity are presented, showing both the orbital phases for each of them. As reported in a recent work by some of us [52], the reactivity of phenol is described by a CPET mechanism since the transferred electron belongs to a π spin IBO of the aromatic system. Conversely, the β spin IBO of the phenolic O–H σ bond remains localized on the same molecule. Thus, the proton and the electron travel separately. Here, we show that when oxygen is replaced by any other chalcogen, the mechanism becomes HAT. Figure 2 shows the spin IBOs for the reaction with phenyl thiol, but analogous results are computed in the case of Se and Te (Figure S1). Particularly, the HAT mechanism is revealed by the modification of a β spin IBO (blue) which is initially part of the S–H σ bond and progressively becomes part of the newly formed O–H σ bond of the hydrogen peroxide. In each step of the reaction path, this spin IBO surrounds the transferred proton, maintaining the σ symmetry. Therefore, the proton and the electron travel together as in a HAT mechanism.

To complete this picture, the corresponding α spin IBO (green) of the S–H σ bond remains localized on the aromatic moiety, particularly on the sulfur atom, determining the radical character of the product. Lastly, the α’ spin IBO (purple) localized on the peroxyl moiety becomes part of the newly formed O–H σ bond, pairing the electron in the hydrogen peroxide. Similarly, in the Se and Te systems, the unpaired α electron is localized on the chalcogen atom. Conversely, the CPET mechanism from the π system of phenol leaves an electron belonging to the aromatic system unpaired.

**Figure 3:**
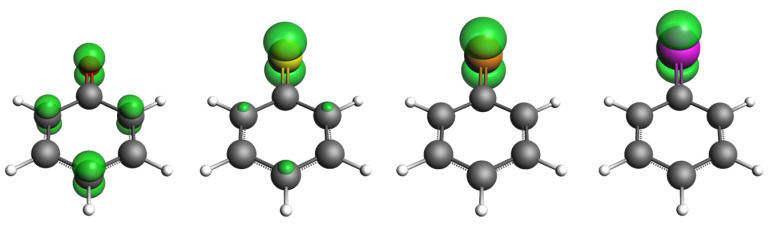
Spin densities on the phenyl chalcogenyl radical (PhX^•^); X = O, S, Se and Te, respectively. Isosurface value of 0.016. Level of theory: ZORA-M06-2X/TZ2P//M06-2X/6-31G(d),cc-PVTZ-pp.

The mechanism changes upon replacing oxygen with a different chalcogen is also supported by the radical character of the radical products and, particularly, by their spin densities shown in Figure 3. Since phenyl selenol and tellurol are characterized by two lower-lying electronic states [60], the most stable one (π-state) was considered for computing the spin densities. In the presence of oxygen, the spin is delocalized all over the ring and the heteroatom, supporting a CPET mechanism from the π system. When increasing the size of the chalcogen, the spin delocalization on the aromatic ring decreases and the radical character becomes localized on the heteroatom. This is consistent with the HAT mechanism, in which only the chalcogen is involved in the reactivity.

### 2.2. Aromaticity Role in ROS Scavenging Activity and Mechanism of Chalcogenols

To rationalize the role of aromatic moieties as well as the chalcogen effect on the hydrogen abstraction mechanisms, two well-known reactions are considered: (I) the phenol/phenoxyl self-exchange reaction, also extended to S, Se and Te derivatives, to evaluate the role of the aromaticity in a symmetric system; (II) the methanol/methoxyl self-exchange reaction, also extended to S, Se and Te derivatives, to analyze the chalcogen effect in the absence of aromatic moieties.

Starting with the systems of case (I), each self-exchange reaction is thermoneutral since the reactants and the products are the same, i.e., a phenyl chalcogenol (PhXH) and the corresponding radical (PhX^•^). Indeed, no mixed system is included in the analysis. Therefore, the feasibility of these reactions relies on the kinetics. Transition state energies in the gas phase, water and benzene are shown in Table 3. As in the previous reaction, both electronic and Gibbs free activation energies decrease when heavier chalcogens are included, regardless of the solvent. Particularly, electronic energies become slightly negative in the case of Te due to the presence of a stabilized reactant complex, whose inclusion would allow to recover an energy barrier. For an accurate description of this issue the readers should refer to the outstanding review article by Bickelhaupt and coworkers [61]. However, this interacting system no longer exists on the PES of Gibbs free energy due to entropy destabilization. Thus, the computed Gibbs free activation energies well describe the kinetics of these processes. In the gas phase, the highest barrier is 12.3 kcal mol^−1^ (phenol systems), while the smallest one is 9.7 kcal mol^−1^ (tellurol system). Furthermore, the energy barrier values increase when solvation is included, especially with lighter chalcogens; particularly, the effect is more pronounced increasing the polarity of the environment.

**Figure 4:**
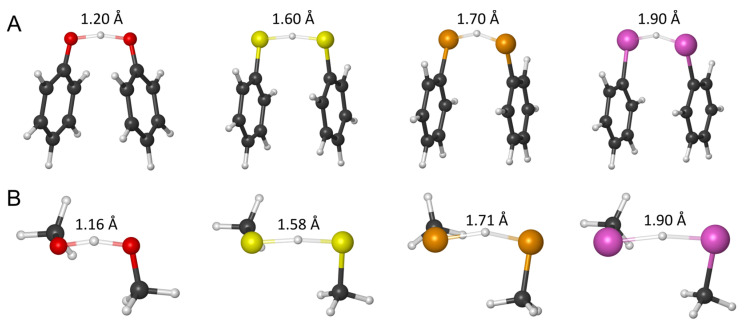
Fully optimized structure of the transition states for (**A**) PhXH/PhX^•^ and (**B**) MeXH/MeX^•^ self-exchange reactions. Symmetric X–H distances are reported in Å. Level of theory: M06-2X/6-31G(d),cc-PVTZ-pp.

Figure 4A shows the fully optimized structures of the transition states for this process. Since the system is symmetric, both X–H bond lengths are equal for each structure, with increasing distances for heavier chalcogens, due to the atom size. A particularly high X–H bond elongation is computed when moving form O to S (0.4 Å), while S and Se are characterized by the shortest increment (0.1 Å). These distances may have an effect on the reaction mechanism involved, suggesting a greater difference between O and S.

Like in the previous reactions, the radical product is PhX^•^ with the spin density shown in Figure 3. Hence, the participation of the π system in the reactivity is expected only when oxygen is involved, since the spin delocalization on the ring is negligible in presence of the other chalcogens. Indeed, the phenol/phenoxyl self-exchange reaction is described by a CPET mechanism [62,63,64,65], with the electron transfer occurring between the two π systems [52]. Conversely, the active mechanism in the other processes is analyzed by looking at the electron flow during the reaction though the involved IBOs. Similar results are obtained for S, Se and Te, showing HAT is the mechanism in each case. Particularly, the β spin IBO of the σ X–H bond in one molecule becomes part of the newly formed σ X–H bond in the other molecule (Figures S2 and S3), following the proton at each step of the reaction path. Therefore, the mechanism switches from CPET in the phenol/phenoxyl system to HAT when oxygen is replaced by any heavier chalcogen.

Finally, the system of case (II), i.e., the self-exchange reaction between methyl chalcogenols and their corresponding radicals, is evaluated. Also in this case, being an exchange reaction, no thermodynamic data is relevant to have insight into the reactivity. The transition state energies are reported in Table 4, showing that the energy barrier decreases as the chalcogen size increases, similarly to the previously observed trends. However, the barriers in presence of S and Se are almost identical and, based on the Gibbs free energies in water, the activation energy is slightly lower in presence of S. Still, the highest energy barrier is computed when oxygen is included and the lowest one is computed for Te, regardless of the solvent.

Transition state geometries are reported in Figure 4B also showing the differences with the other self-exchange processes. Similarly to the previous case, the X–H bond lengths are equal within the same structure, due to the symmetry of the reaction. Furthermore, each X–H distance is very close to the one of the corresponding aromatic TS; thus, the same considerations are valid.

The absence of an aromatic moiety simplifies the understanding of the reaction mechanism. Indeed, all MeXH/MeX^•^ self-exchange reactions are found to be HAT, consistently with previous analysis on the methanol/methoxyl system [52,62]. Particularly, no other electron could be transferred between the two molecules rather than the one localized on the X–H σ bond. Therefore, the proton and the electron must travel together.

## 3. Discussion

To rationalize the kinetic trend and the mechanism change in each process, ASA is performed on the four chalcogen-systems along the reaction path, starting from the quenching of ^•^OOH by phenyl chalcogenols. The decomposition of the total energy in ${∆E}_{strain}$ and ${∆E}_{int}$ is computed along a specific reaction coordinate, which is the X–H bond breaking, i.e., the distance between the chalcogen X and the transferred H with respect to their initial distance in the reactant. This reaction coordinate is chosen to maintain consistency between different chalcogens and their initial X–H bond length.

**Figure 5:**
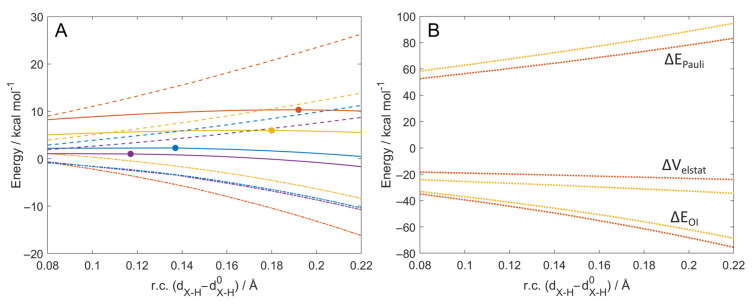
(**A**) Activation strain analysis of the hydrogen abstraction from phenyl chalcogenol (PhXH) by ^•^OOH: energy profiles (solid lines), ${∆E}_{strain}$ (dashed lines), ${∆E}_{int}$ (dash-dotted lines) along the reaction path for X = O (red), S (yellow), Se (blue) and Te (purple). The filled circles represent the position of the transition states. (**B**) Energy decomposition analysis (dotted lines): ${∆E}_{Pauli}$, ${∆V}_{elstat}$, ${∆E}_{OI}$. The reaction coordinate is defined as r.c. = (d_X–H_ − d_X–H_^0^), where d_X–H_^0^ represents the X–H bond distance in the reactant of each reaction. Level of theory: ZORA-M06-2X/TZ2P//M06-2X/6-31G(d),cc-PVTZ-pp.

Figure 5A shows the energy profiles of the four reactions, highlighting the decrease of the energy barrier when going down along the chalcogen group. ${∆E}_{strain}$ reproduces the kinetic trend, showing, for the O based system, the highest strain, which progressively decreases for S, Se and Te based systems. Since ^•^OOH is consistent in each reaction, the ${∆E}_{strain}$ differences are mainly attributed to the deformation of phenyl chalcogenols. Conversely, ${∆E}_{int}$ is not consistent with the kinetic trend, particularly when oxygen is present, since it is characterized by the most favorable interaction. Therefore, the kinetic trend is strain-controlled and a bigger chalcogen size reflects a lower destabilization in the X–H elongation for the hydrogen abstraction. Indeed, Te has a soft and polarizable character, and better responds to chemical perturbations, which justifies the lowest energy barrier.

Despite ${∆E}_{strain}$ explaining the energy trend, it does not rationalize the change in mechanism. Thus, ${∆E}_{int}$ and its decomposition may be insightful when comparing oxygen and sulfur, since a greater stabilizing interaction is computed for oxygen despite its higher activation energy. Particularly, ${∆E}_{int}$ (and especially ${∆E}_{OI}$) is reported to be a fundamental factor to allow a CPET mechanism [52]. Figure 5B shows the energy contributions to ${∆E}_{int}$ limited to these two systems for clarity. This analysis reveals a more stabilizing ${∆E}_{OI}$ and a less destabilizing ${∆E}_{Pauli}$ in the case of oxygen, determining the more favorable interaction energies. Conversely, ${∆V}_{elstat}$ tends to stabilize the process with the phenyl thiol, without prevailing. Therefore, the switch from the CPET mechanism of phenol to the HAT mechanism of phenyl thiol occurs in correspondence with lower orbital contributions to the interaction energy, which are reported to be significant in CPET reactions [52].

**Figure 6:**
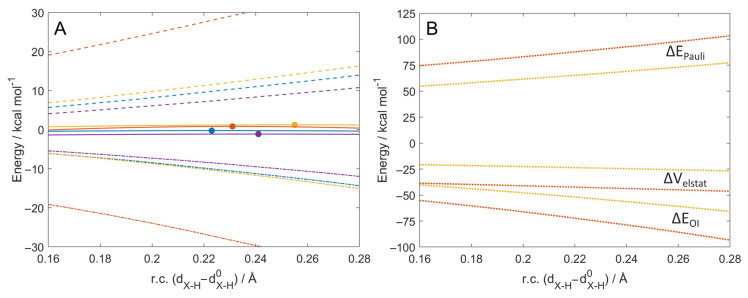
(**A**) Activation strain analysis of PhXH/PhX^•^ self-exchange reactions: energy profiles (solid lines), ${∆E}_{strain}$ (dashed lines), ${∆E}_{int}$ (dash-dotted lines) along the reaction path for X = O (red), S (yellow), Se (blue) and Te (purple). The filled circles represent the position of the transition states. (**B**) Energy decomposition analysis (dotted lines): ${∆E}_{Pauli}$, ${∆V}_{elstat}$, ${∆E}_{OI}$. The reaction coordinate is defined as r.c. = (d_X–H_ − d_X–H_^0^), where d_X–H_^0^ represents the X–H bond distance in the reactant of each reaction. Level of theory: ZORA-M06-2X/TZ2P//M06-2X/6-31G(d),cc-PVTZ-pp.

Moving to PhX/PhX^•^ self-exchange reactions (case(I)), activation strain analysis is employed to rationalize the kinetic results. The chosen reaction coordinate is the X–H bond breaking to maintain consistency with the previous analysis. Figure 6A shows the electronic energy profile of the four reactions in the gas phase. Hence, oxygen and sulfur curves are almost superimposed with very close energy barriers, as reported in Table 3. In fact, it is the inclusion of entropy and/or solvation effects that differentiate the two energies, leading to the above-described kinetic trend (O > S > Se > Te). Again, the reactivity is strain-controlled, since ${∆E}_{strain}$ reproduces the energy trend in presence of all chalcogens, except oxygen. In fact, ${∆E}_{strain}$ would determine a very high energy barrier for the phenol/phenoxyl system; however, a strongly stabilizing ${∆E}_{int}$ is also computed for this system, making the total energy comparable to the one of sulfur system. Overall, the lower strain in presence of the heavier chalcogens explains their higher reactivity, but a significant stabilizing interaction is likely the cause of the CPET mechanism when oxygen is present.

The energy decomposition analysis (Figure 6B) indicates ${∆V}_{elstat}$ and ${∆E}_{OI}$ as the responsible contributions to the higher stabilization of ${∆E}_{int}$ in presence of oxygen rather than sulfur. Hence, only the phenol/phenoxyl system seems to be characterized by a sufficiently stabilizing orbital interaction to allow the participation of the aromatic moiety in the reactivity though a CPET mechanism. Conversely, the loss of ${∆E}_{OI}$ may be responsible for the mechanism switch to HAT, when the heavier chalcogens are present.

Finally, despite the mechanism switch in case (II) does not occur, ASA (Figure S4) is used to rationalize the kinetic trends and the chalcogen effect on the reactivity in MeX/MeX^•^ self-exchange reactions. Analogously to the other cases, the reaction is strain-controlled; a lower ${∆E}_{strain}$ is observed in presence of the heavier chalcogens, reproducing the energy trend. Particularly, S and Se curves are superimposed in both ${∆E}_{strain}$ and ${∆E}_{int}$, showing the similar role of the two chalcogens in this reaction.

Overall, the absence of an aromatic system homologates the reaction mechanisms in presence of all the different chalcogens, while the chalcogen effect on the process becomes less evident (especially for S and Se) without significantly altering the kinetic trend. Indeed, this effect is strain-controlled, regardless of the active mechanism; therefore, no remarkable change in the overall energetics was computed.

## 4. Materials and Methods

Density Functional Theory (DFT) calculations were carried out using Gaussian16 [66]. The M06-2X [67] functional was used for all geometry optimizations, combined with the 6-31G(d) basis set for each atom with the exception of Te, for which the cc-PVTZ basis set was used including the small-core pseudo potential of Lim et al. [68]. Frequency calculations were performed for all optimized structures to extract thermodynamic corrections and to assess the nature of each stationary point. Particularly, all minima have real frequencies, while transition states have one imaginary frequency. The reaction paths were calculated using the Intrinsic Reaction Coordinate (IRC) method [69]. The intrinsic bond orbital (IBO) [70,71] method was used to analyze the electron flow along the reaction coordinate, as implemented in IboView software v20211019-RevA. However, this software does not support the 6-31G basis set family; thus, single point calculations were carried out using the def2TZVP basis set for each atom. Spin contamination was checked for all doublet species and was found to be negligible. The overall level of theory was chosen in agreement with the QM-ORSA protocol for the calculation of radical scavenging potential [14,72].

Activation Strain Analysis (ASA) and Energy Decomposition Analysis (EDA) were performed along a selected reaction coordinate [73,74,75,76], using IRC profiles and the program PyFrag 2019 [77]. To be consistent with the analysis and to obtain more accurate estimates of the electronic energies, single point calculations were performed using Amsterdam Density Functional (ADF) 2019.307 [78,79]. The zeroth-order regular approximation (ZORA) was employed to include scalar relativistic effects, fundamental for heavy atoms like Te [80]; in order to maintain consistency within the work, ZORA was employed for every calculation, regardless of the chalcogen, as it is commonly done with great performances [81,82,83]. The same functional used for the optimization procedure was employed together with the all electron TZ2P basis set for all atoms. Solvation effects were also included in single point calculations by using the conductor-like screening model (COSMO) [84]. Particularly, water and benzene solvents were chosen to simulate polar and non-polar environments, respectively; in agreement with the scavenging potential analysis performed on small organic molecules [14,46,47,48,49,85,86] (level of theory: (COSMO)-ZORA-M06-2X/TZ2P//M06-2X/6-31G(d),cc-PVTZ-pp).

ASA is a fragment-based approach relying on the definition of chemically meaningful fragments. In this method, the total energy is expressed as the sum of two contributions at any point along the reaction coordinate (ζ):

(1) $$∆E(\zeta )={∆E}_{strain}(\zeta )+{∆E}_{int}(\zeta ),$$

where ${∆E}_{strain}$ represents the energy required to distort the relaxed fragments to obtain the proper structure at a specific reaction coordinate, and ${∆E}_{int}$ is the actual interaction energy between these distorted fragments. Moreover, this latter term can be split into different contributions, in the framework of EDA:

(2) $${∆E}_{int}\left(\zeta \right)={∆V}_{elstat}\left(\zeta \right)+{∆E}_{OI}\left(\zeta \right)+{∆E}_{Pauli}\left(\zeta \right)+{∆E}_{disp}\left(\zeta \right),$$

where ${∆V}_{elstat}$ is the semiclassical electrostatic interaction between the unperturbed electron densities of the distorted fragments; ${∆E}_{OI}$ accounts for all the occupied-void orbital interactions; ${∆E}_{Pauli}$ (Pauli or exchange repulsion) represents the repulsion between occupied orbitals localized on the two fragments, and ${∆E}_{disp}$ accounts for dispersive interactions, within the model of dispersion used in the calculations.

## 5. Conclusions

The ROS scavenging activity and mechanism of phenyl chalcogenols were evaluated in silico to highlight the role of the chalcogen. Hydrogen abstraction by ^•^OOH is thermodynamically and kinetically more efficient with heavier chalcogens due to a decrease in the X–H bond strength when moving down along group 16. Furthermore, the presence of different chalcogens affects the reaction mechanism. The reactivity of phenol is described by a CPET mechanism with the involvement of the aromatic moiety in the electron transfer; whereas HAT occurs when the oxygen atom is replaced by the other chalcogens. Hence, the aromatic system is no longer involved in the reactivity. The kinetic trend is rationalized in the framework of activation strain analysis, showing a lower energy strain in breaking the X–H bond with heavier chalcogen due to their soft character. Conversely, the mechanism switch from CPET to HAT occurs in correspondence of a significant decrease in the orbital interaction contribution to the total energy.

Lastly, the importance of the aromatic moiety is evaluated in the phenol/phenoxyl and methanol/metoxyl self-exchange reactions, as well as in the corresponding processes with the other chalcogens. The kinetic trend remains consistent with lower energy barriers for heavier chalcogens; however, the energy differences become less significant in the absence of aromatic moieties. Moreover, the mechanism switch from CPET to HAT still occurs in the aromatic system when replacing the oxygen atom with its siblings of group 16. Conversely, the absence of aromatic structures does not allow a CPET mechanism; therefore, only HAT is effective, regardless of the involved chalcogen atom.

This analysis gives further insight into the chalcogen role in the ROS scavenging potential of small organic molecules from an energy and mechanistic point of view. Particularly, CPET or HAT emerges as the effective mechanism depending on the chemical topology.

## Figures and Tables

**Table 1:** Electronic (ΔE) and Gibbs free (ΔG°) reaction energies (kcal mol^−1^) for the hydrogen abstraction from phenyl chalcogenols by ^•^OOH in the gas phase, water and benzene. Level of theory: (COSMO)-ZORA-M06-2X/TZ2P//M06-2X/6-31G(d),cc-PVTZ-pp.

X	ΔE			ΔG°		
	Gas	Water	Benzene	Gas	Water	Benzene
O	4.5	0.8	2.9	3.9	1.6	2.3
S	−7.5	−8.6	−8.1	−5.2	−6.5	−5.8
Se	−18.4	−19.9	−19.2	−15.6	−17.1	−16.4
Te	−29.1	−30.7	−30.0	−25.2	−26.8	−26.1

**Table 2:** Electronic (ΔE^‡^) and Gibbs free (ΔG^‡^) activation energies (kcal mol^−1^) for the hydrogen abstraction from phenyl chalcogenols by ^•^OOH in the gas phase, water and benzene, with respect to free reactants. Level of theory: (COSMO)-ZORA-M06-2X/TZ2P//M06-2X/6-31G(d),cc-PVTZ-pp.

X	ΔE^‡^			ΔG^‡^		
	Gas	Water	Benzene	Gas	Water	Benzene
O	10.8	12.4	12.1	18.6	20.3	19.9
S	6.4	9.4	8.0	16.0	19.1	17.6
Se	2.7	5.3	4.1	12.9	15.5	14.3
Te	1.4	3.9	2.8	11.8	14.3	13.2

**Table 3:** Electronic (ΔE^‡^) and Gibbs free (ΔG^‡^) activation energies (kcal mol^−1^) for PhXH/PhX^•^ self-exchange reactions in the gas phase, water and benzene, with respect to free reactants. Level of theory: (COSMO)-ZORA-M06-2X/TZ2P//M06-2X/6-31G(d),cc-PVTZ-pp.

X	ΔE^‡^			ΔG^‡^		
	Gas	Water	Benzene	Gas	Water	Benzene
O	1.6	5.5	3.6	12.3	16.2	14.3
S	2.0	3.0	2.5	11.4	12.4	11.9
Se	0.4	0.9	0.7	10.7	11.2	11.0
Te	−0.4 *	−0.2 *	−0.3 *	9.6	9.8	9.7

* Negative values due to the presence of a stabilized reactant complex. Energy barriers are 6.6 (gas), 6.5 (water) and 6.6 (benzene) kcal mol^−1^.

**Table 4:** Electronic (ΔE^‡^) and Gibbs free (ΔG^‡^) activation energies (kcal mol^−1^) for MeXH/MeX^•^ self-exchange reactions in the gas phase, water and benzene, with respect to free reactants. Level of theory: (COSMO)-ZORA-M06-2X/TZ2P//M06-2X/6-31G(d),cc-PVTZ-pp.

X	ΔE^‡^			ΔG^‡^		
	Gas	Water	Benzene	Gas	Water	Benzene
O	7.7	11.7	9.7	15.9	19.9	17.9
S	3.9	3.0	6.5	11.7	10.9	14.4
Se	3.4	3.1	5.9	12.0	11.8	14.5
Te	1.6	0.6	3.6	9.7	8.6	11.6

## Data Availability

All data used are available in the main text or in the Supplementary Material.

## Supplementary Materials

The following supporting information can be downloaded at: https://www.mdpi.com/article/10.3390/molecules30071408/s1, Figure S1. Changes in the main spin IBO involved in the hydrogen abstraction from phenyl selenols/tellurol by ^•^OOH along the reaction path: β spin IBO transferred from the XH σ bond to the other molecule. Level of theory: M06-2X/def2TZVP//M06-2X/6-31G(d),cc-PVTZ-pp; Figure S2: Changes in the main spin IBOs involved in PhSH/PhS^•^ self-exchange reactions along the reaction path: β spin IBO (blue) transferred from the SH σ bond to the other molecule, the corresponding α spin IBO (green), and the S-centered α′ spin IBO (purple) of the radical. Analogous IBOs are found for Se and Te compounds. Level of theory: M06-2X/def2TZVP//M06-2X/6-31G(d); Figure S3. Changes in the main spin IBO involved in PhSeH/PhSe^•^ and PhTeH/PhTe^•^ self-exchange reactions along the reaction path: β spin IBO transferred from the XH σ bond to the other molecule. Level of theory: M06-2X/def2TZVP//M06-2X/6-31G(d),cc-PVTZ-pp; Figure S4: Activation strain analysis of MeXH/MeX^•^ self-exchange reactions: energy profiles (solid lines), ${∆E}_{strain}$ (dashed lines), ${∆E}_{int}$ (dash-dotted lines) along the reaction path for X = O (red), S (yellow), Se (blue) and Te (purple). The filled circles represent the position of the transition states. The reaction coordinate is defined as r.c. = (d_X–H_ − d_X–H_^0^), where d_X–H_^0^ represents the X–H bond distance in the reactant of each reaction. Level of theory: ZORA-M06-2X/TZ2P//M06-2X/6-31G(d),cc-PVTZ-pp; Table S1: Coordinates (Å) and energies (E, Hartree) of stationary points, number of imaginary frequencies (Nimag, cm^−1^) of transition states, S^2^ eigenvalue and spin contamination (S^2^_err%) for all doublet species. Level of theory: M06-2X/6-31G(d),cc-PVTZ-pp.

## Abbreviations

The following abbreviations are used in this manuscript:

ADF	Amsterdam Density Functional
ASA	Activation Strain Analysis
BDE	Bond Dissociation Enthalpy
COSMO	COnductor-like Screening MOdel
CPET	Concerted Proton Electron Transfer
DFT	Density Functional Theory
EDA	Energy Decomposition Analysis
GPx	Glutathione Peroxidases
HAT	Hydrogen Atom Transfer
IBO	Intrinsic Bond Orbital
IRC	Intrinsic Reaction Coordinate
SOD	SuperOxide Dismutase
SPLET	Sequential Proton Loss Electron Transfer
ZORA	Zeroth Order Regular Approximation
