# Supplementary material for: The Role of Chalcogen in the ROS Scavenging Mechanism of Model Phenyl Compounds
Source: Molecules. 2025 Mar 21;30(7):1408. doi: 10.3390/molecules30071408 (PMC11990681; doi:10.3390/molecules30071408)
Supplement: Supplementary file 1 [file molecules-30-01408-s001.zip › molecules-3536726-supplementary.pdf]

# – Supplementary Material –

The Role of Chalcogen in the ROS Scavenging Mechanism of Model Phenyl Compounds

Davide Zeppilli [1], Veronica Pedergnana [1], Matteo Filippi [1] and Laura Orian [1,*]

[1] Dipartimento di Scienze Chimiche, Università degli Studi di Padova, Via Marzolo 1, 35131 Padova, Italy
[*] Correspondence: laura.orian@unipd.it

LIST of TABLES and FIGURES:

Figure S1. Changes in the main spin IBO involved in the hydrogen abstraction from phenyl selenols/tellurol by •OOH along the reaction path: β spin IBO transferred from the XH σ bond to the other molecule. Level of theory: M06-2X/def2TZVP//M06-2X/6-31G(d),cc-PVTZ-pp……………………………………………...S2

Figure S2. Changes in the main spin IBOs involved in PhSH/PhS• self-exchange reactions along the reaction path: β spin IBO (blue) transferred from the SH σ bond to the other molecule, the corresponding α spin IBO (green), and the S-centered α' spin IBO (purple) of the radical. Analogous IBOs are found for Se and Te compounds. Level of theory: M06-2X/def2TZVP//M06-2X/6-31G(d)….…………..………………………S2

Figure S3. Changes in the main spin IBO involved in PhSeH/PhSe• and PhTeH/PhTe• self-exchange reactions along the reaction path: β spin IBO transferred from the XH σ bond to the other molecule. Level of theory: M06-2X/def2TZVP//M06-2X/6-31G(d),cc-PVTZ-pp……………………………………………...S3

Figure S4. Activation strain analysis of MeXH/MeX• self-exchange reactions: energy profiles (solid lines), ΔE_{strain} (dashed lines), ΔE_{int} (dash-dotted lines) along the reaction path for X= O (red), S (yellow), Se (blue) and Te (purple). The filled circles represent the position of the transition states. The reaction coordinate is defined as r.c.= ($d_{X\text{-}H}$ – $d_{X\text{-}H}^0$), where $d_{X\text{-}H}^0$ represents the X–H bond distance in the reactant of each reaction. Level of theory: ZORA-M06-2X/TZ2P//M06-2X/6-31G(d),cc-PVTZ-pp………………………………………S3

Table S1. Coordinates (Å) and energies (E, Hartree) of stationary points, number of imaginary frequencies (Nimag, cm^{-1}) of transition states, S^2 eigenvalue and spin contamination (S^2_err%) for all doublet species. Level of theory: M06-2X/6-31G(d),cc-PVTZ-pp……………………………………………….…………S4

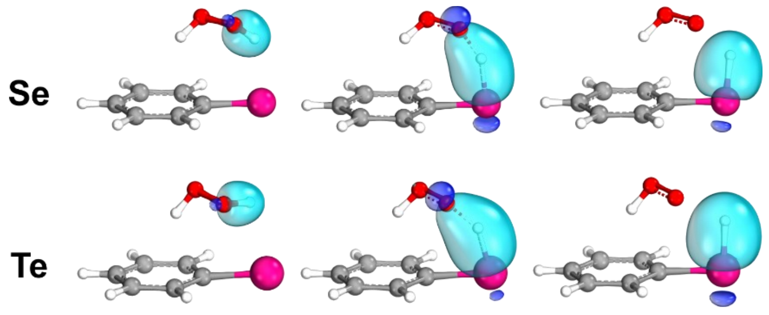

Figure S1. Changes in the main spin IBO involved in the hydrogen abstraction from phenyl selenols/tellurol by $^{\bullet}$OOH along the reaction path: β spin IBO transferred from the XH σ bond to the other molecule. Level of theory: M06-2X/def2TZVP//M06-2X/6-31G(d),cc-PVTZ-pp.

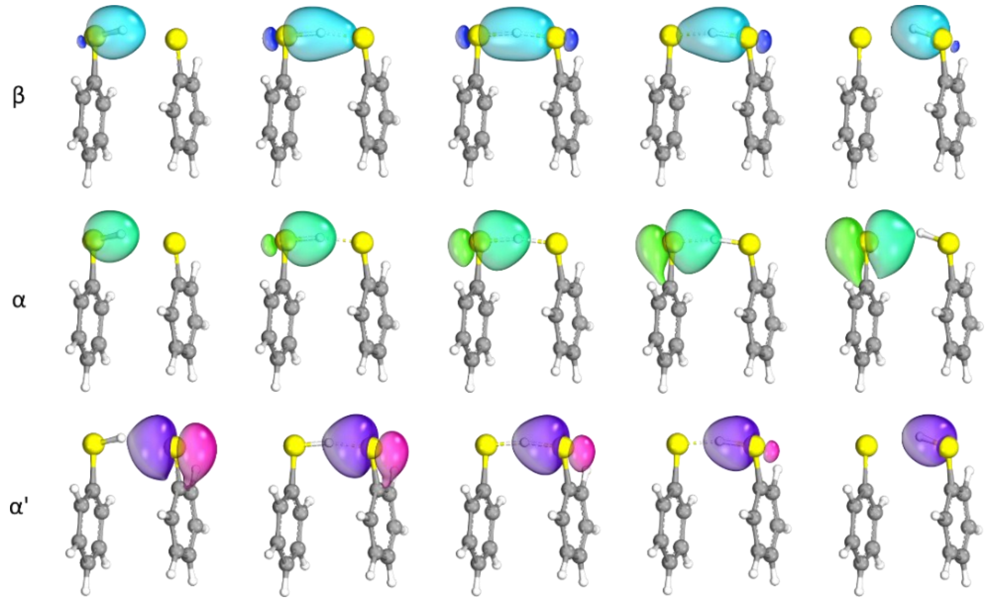

Figure S2. Changes in the main spin IBOs involved in PhSH/PhS$^{\bullet}$ self-exchange reactions along the reaction path: β spin IBO (blue) transferred from the SH σ bond to the other molecule, the corresponding α spin IBO (green), and the S-centered α' spin IBO (purple) of the radical. Analogous IBOs are found for Se and Te compounds. Level of theory: M06-2X/def2TZVP//M06-2X/6-31G(d),cc-PVTZ-pp.

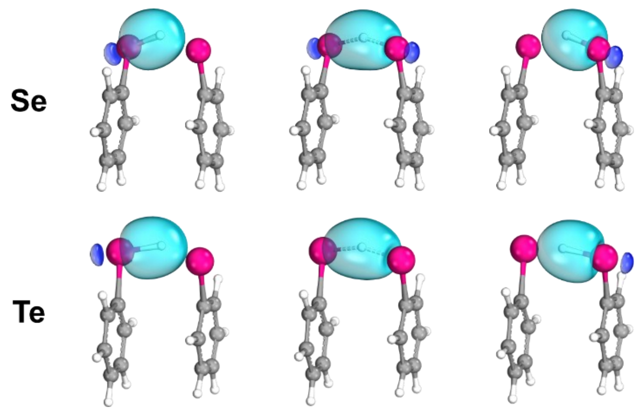

Figure S3. Changes in the main spin IBO involved in PhSeH/PhSe• and PhTeH/PhTe• self-exchange reactions along the reaction path: β spin IBO transferred from the XH σ bond to the other molecule. Level of theory: M06-2X/def2TZVP//M06-2X/6-31G(d),cc-PVTZ-pp.

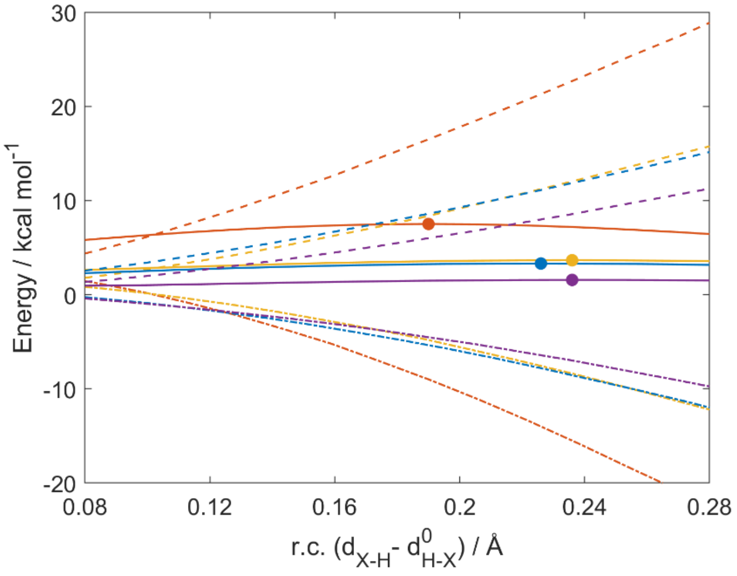

Figure S4. Activation strain analysis of MeXH/MeX• self-exchange reactions: energy profiles (solid lines), ΔE_{strain} (dashed lines), ΔE_{int} (dash-dotted lines) along the reaction path for X= O (red), S (yellow), Se (blue) and Te (purple). The filled circles represent the position of the transition states. The reaction coordinate is defined as r.c.= (d_{X-H} – d_{X-H}^0), where d_{X-H}^0 represents the X–H bond distance in the reactant of each reaction. Level of theory: ZORA-M06-2X/TZ2P//M06-2X/6-31G(d).

Table S1. Coordinates (Å) and energies (E, Hartree) of stationary points, number of imaginary frequencies (Nimag, cm^{-1}) of transition states, S^2 eigenvalue and spin contamination (S^2_err%) for all doublet species. Level of theory: M06-2X/6-31G(d),cc-PVTZ-pp.

PhXH + $^{\bullet}$OOH

$^{\bullet}$OOH E= -150.832724

S^2= 0.7500 S^2_err%= 0%

O	0.055503	0.709875	0.000000
O	0.055503	-0.601901	0.000000
H	-0.888054	-0.863793	0.000000

HOOH E= -151.4681908

O	0.000000	0.713705	-0.055572
O	0.000000	-0.713705	-0.055572
H	-0.812252	-0.891397	0.444578
H	0.812252	0.891397	0.444578

PhOH E= -307.329665839

C	1.459724	-0.002560	-1.590836
C	0.302766	-0.357463	-0.902738
C	2.613494	0.351389	-0.898429
H	-0.598328	-0.633926	-1.445875
H	3.513008	0.627411	-1.438130
C	0.300782	-0.358135	0.491987
C	2.600694	0.347461	0.495887
H	3.494895	0.621866	1.047414
C	1.452993	-0.004672	1.194890
H	1.424902	-0.013201	2.279137
H	1.453956	-0.004310	-2.676608
O	-0.797984	-0.695042	1.222970
H	-1.523307	-0.915719	0.622098

PhO$^{\bullet}$ E= -306.686310713

S^2= 0.7511 S^2_err%= 0.15%

C	0.799615	0.004475	-2.571996
C	-0.367308	-0.250990	-1.894008
C	1.975755	0.312346	-1.863132
H	-1.293812	-0.491755	-2.404912
H	2.892466	0.513271	-2.408071
C	-0.409810	-0.208942	-0.444793
C	1.978709	0.363475	-0.456758
H	2.898800	0.602945	0.067107
C	0.825003	0.112008	0.244990
H	0.787856	0.142112	1.328994
H	0.822951	-0.029247	-3.656719
O	-1.465645	-0.439057	0.182761

TS(O) E= -458.147730965

S^2= 0.7502 S^2_err%= 0.03%

Nimag= -2139.66

O	9.260700	12.992977	0.769349
O	8.040723	12.406342	0.957669
H	9.889566	12.258488	0.896182
C	8.977317	13.329684	3.583297
C	9.687323	12.502222	4.488119
C	9.585580	14.523147	3.118156
H	9.203395	11.592486	4.827806
H	9.029277	15.144210	2.424601
C	10.944196	12.872955	4.926149
C	10.854071	14.873702	3.556595
H	11.480267	12.244915	5.630790
H	11.315112	15.788826	3.199207
C	11.533979	14.056553	4.459716
H	12.523470	14.338140	4.805600
O	7.766086	12.990930	3.203845
H	7.737715	12.801439	2.057462

PhSH E= -630.288593574

C	1.493672	0.008108	-1.594557
C	0.347321	-0.342639	-0.887673
C	2.658204	0.364630	-0.921191
H	-0.555572	-0.618783	-1.424908
H	3.549656	0.637445	-1.476213
C	0.360233	-0.338621	0.508176
C	2.668674	0.368002	0.471529
H	3.57062	0.644070	1.009059
C	1.528529	0.019017	1.186261
H	1.543117	0.023381	2.272402
H	1.471511	0.001258	-2.680043
S	-1.059550	-0.772341	1.484765
H	-1.878824	-1.030429	0.454158

PhS$^{\bullet}$ E= -629.663316201

S^2= 0.7501 S^2_err%= 0.01%

C	0.838804	0.013662	-2.581178
C	-0.333932	-0.242790	-1.888408
C	2.009300	0.319409	-1.883024
H	-1.251804	-0.481917	-2.415542
H	2.926476	0.519887	-2.428280
C	-0.357373	-0.198000	-0.475904
C	2.006166	0.368955	-0.487137
H	2.918589	0.607381	0.049968
C	0.837110	0.113863	0.212268
H	0.817460	0.148578	1.296645
H	0.847255	-0.023047	-3.665809
S	-1.813470	-0.515340	0.389862

TS(S) E= -781.112930767

S^2= 0.7501 S^2_err%= 0.01%

Nimag= -1688.05

O	9.554898	12.533497	1.031115
O	8.449716	11.791468	1.229250
H	10.133368	12.311397	1.790590
C	8.867560	13.6355290	3.606823
C	9.583371	12.609910	4.246818
C	9.521434	14.835647	3.296514
H	9.071890	11.683384	4.489749
H	8.969538	15.617303	2.784985
C	10.929567	12.781955	4.554494

C	10.861514	15.011627	3.629108
H	11.477876	11.981943	5.042343
H	11.358089	15.945901	3.387125
C	11.569202	13.985070	4.250323
H	12.617559	14.119138	4.497486
S	7.192978	13.374832	3.113506
H	7.670216	12.498415	1.954314

PhSeH E= -2631.55594811

C	1.530254	0.120522	-1.664258
C	0.315051	-0.093004	-1.019050
C	2.694873	0.291486	-0.919815
H	-0.599796	-0.224150	-1.587727
H	3.641478	0.460012	-1.423821
C	0.266188	-0.140466	0.373261
C	2.644228	0.250341	0.471295
H	3.549496	0.385925	1.054902
C	1.430360	0.036232	1.119157
H	1.377634	0.004210	2.202535
H	1.565561	0.154752	-2.748730
Se	-1.411683	-0.402167	1.264489
H	-1.306050	-1.880595	1.279528

PhSe$^{\bullet}$ E= -2630.92970244

S^2= 0.7500 S^2_err%= 0%

C	0.853017	0.017216	-2.578942
C	-0.324730	-0.240139	-1.884651
C	2.025289	0.322873	-1.892531
H	-1.235003	-0.478057	-2.427869
H	2.941898	0.522848	-2.437400
C	-0.335115	-0.192663	-0.489256
C	2.011395	0.369836	-0.500969
H	2.918122	0.607157	0.046986
C	0.838535	0.114044	0.202070
H	0.836137	0.152804	1.287764
H	0.849612	-0.022650	-3.663899
Se	-1.934576	-0.542626	0.462158

TS(Se) E= -2782.38657076

S^2= 0.7501 S^2_err%= 0.01%

Nimag= -1418.73

O	9.559535	12.608645	1.025987
O	8.503003	11.801737	1.184432
H	10.130345	12.416488	1.800204
C	8.876824	13.626269	3.655621
C	9.593713	12.607375	4.295528
C	9.527236	14.814728	3.309432
H	9.078719	11.689253	4.5602590
H	8.967911	15.588647	2.794456
C	10.948966	12.773532	4.570925
C	10.877178	14.985997	3.605236
H	11.501875	11.977002	5.059231
H	11.375067	15.911442	3.333654
C	11.590533	13.964820	4.229251
H	12.645609	14.094450	4.448828
Se	7.068422	13.344637	3.158925
H	7.583843	12.511994	1.872574

PhTeH E= -500.065007136

C	1.528113	0.015232	-1.603020
C	0.402154	-0.353718	-0.869887
C	2.697304	0.391638	-0.946988
H	-0.508075	-0.646230	-1.383198
H	3.573147	0.679786	-1.519850
C	0.446066	-0.350279	0.525927
C	2.740859	0.400879	0.444869
H	3.649617	0.695681	0.960394
C	1.617304	0.034077	1.182500
H	1.652390	0.045040	2.267031
H	1.489149	0.008487	-2.687878
Te	-1.278317	-0.874940	1.645614
H	-0.869455	-2.483970	1.751165

PhTe$^{\bullet}$ E= -499.468714419

S^2= 0.7501 S^2_err%= 0.01%

C	0.866621	0.019850	-2.592114
C	-0.309986	-0.237296	-1.895704
C	2.036593	0.325549	-1.899305
H	-1.220942	-0.475177	-2.435605
H	2.953595	0.526007	-2.444525
C	-0.325346	-0.190699	-0.494877
C	2.029302	0.373943	-0.506484
H	2.938957	0.611986	0.035772
C	0.855413	0.117480	0.194755
H	0.850292	0.155201	1.279435
H	0.869262	-0.018591	-3.676867
Te	-2.099180	-0.577611	0.558979

TS(Te) E= -650.894974002

S^2= 0.7501 S^2_err%= 0.01%

Nimag= -1397.61

O	9.667494	12.442878	1.012859
O	8.657561	11.596854	1.225279
H	10.171352	12.432871	1.855482
C	8.882187	13.679726	3.632235
C	9.590332	12.614350	4.2041440
C	9.543745	14.883131	3.371998
H	9.081944	11.677475	4.409760
H	9.007331	15.704565	2.908492
C	10.948547	12.750568	4.491169
C	10.895617	15.022559	3.683796
H	11.492586	11.916792	4.924224
H	11.400215	15.961138	3.477344
C	11.601572	13.956102	4.234446
H	12.657327	14.061313	4.462780
Te	6.878948	13.393167	3.012490
H	7.542752	12.369955	1.734126

PhXH/PhX$^{\bullet}$

TS(O) E= -614.015521288

S^2= 0.7503 S^2_err%= 0.04%

Nimag= -1972.86

C	0.255755	0.785203	2.167383
C	0.514079	-0.565682	2.017184
C	0.066275	1.594561	1.044655

H	0.657257	-1.217776	2.873059
H	-0.134143	2.654131	1.167173
C	0.625937	-1.139881	0.715945
C	0.109383	1.035887	-0.243594
H	-0.061108	1.668455	-1.109500
C	0.392041	-0.302423	-0.414594
H	0.461860	-0.757102	-1.397577
H	0.207056	1.217754	3.161881
O	0.973170	-2.381703	0.552120
H	2.019079	-2.568428	1.112959
C	3.382683	-0.313201	0.728576
C	3.550961	1.048136	0.909655
C	3.319415	-1.189436	1.852630
H	3.563525	1.709312	0.048722
C	3.696284	1.574394	2.195435
C	3.507516	-0.634911	3.153149
H	3.826039	2.643131	2.332730
H	3.475694	-1.316108	3.997451
C	3.700529	0.720748	3.311198
H	3.836519	1.136284	4.305086
H	3.275634	-0.748388	-0.260048
O	3.055039	-2.454157	1.710450

TS(S) E= -1259.95011287

S^2= 0.7502 S^2_err%= 0.03%

Nimag= -1128.36

C	0.360435	-4.071718	-0.472669
C	0.441258	-4.524397	0.838395
C	0.192846	-2.711563	-0.732553
H	0.565736	-5.580425	1.056525
H	0.125837	-2.360695	-1.758063
C	0.358754	-3.614701	1.905763
C	0.110893	-1.801082	0.320944
H	-0.011748	-0.741780	0.117577
C	0.197157	-2.246771	1.633356
H	0.143844	-1.548376	2.462351
H	0.422753	-4.779354	-1.293585
S	0.444347	-4.179358	3.565749
H	2.038046	-4.177324	3.634129
C	3.555255	-2.966789	0.920180
C	3.486154	-1.926762	0.002552
C	3.505310	-2.698216	2.297540
H	3.523272	-2.141882	-1.060875
C	3.358485	-0.610736	0.446855
C	3.375659	-1.369753	2.735850
H	3.303881	0.200862	-0.272576
H	3.337863	-1.169636	3.802069
C	3.300730	-0.334062	1.812789
H	3.202425	0.690792	2.157128
H	3.645988	-3.996093	0.588308
S	3.615447	-4.011635	3.456990

TS(Se) E= -5262.50468424

S^2= 0.7501 S^2_err%= 0.01%

Nimag= -916.32

C	0.385749	-4.307294	-0.299360
C	0.450874	-4.490823	1.076674
C	0.200134	-3.029195	-0.827415
H	0.587356	-5.480059	1.502057
H	0.138247	-2.891361	-1.902662
C	0.359175	-3.386112	1.930577
C	0.101035	-1.930185	0.022299
H	-0.027680	-0.932655	-0.386466
C	0.196967	-2.104161	1.399388
H	0.163371	-1.252423	2.071241
H	0.466627	-5.163933	-0.961438
Se	0.471773	-3.618117	3.800316
H	2.060663	-4.223916	3.698760
C	3.522505	-2.795588	0.683642
C	3.460873	-1.589618	-0.007514
C	3.503137	-2.800345	2.080612
H	3.479427	-1.589870	-1.093049
C	3.345497	-0.390740	0.691889
C	3.394890	-1.594873	2.782895
H	3.284124	0.549131	0.151651
H	3.369902	-1.613562	3.867867
C	3.301635	-0.395858	2.086439
H	3.207900	0.538091	2.632077
H	3.570562	-3.740030	0.151011
Se	3.605885	-4.437959	3.013237

TS(Te) E= -999.536703976

S^2= 0.7504 S^2_err%= 0.05%

Nimag= -900.28

C	0.383029	-4.252485	-0.372356
C	0.383116	-4.502615	0.997655
C	0.243173	-2.949566	-0.846051
H	0.474021	-5.519726	1.365772
H	0.231291	-2.758626	-1.914847
C	0.264130	-3.441054	1.900437
C	0.124510	-1.892141	0.052490
H	0.032154	-0.873518	-0.311994
C	0.148522	-2.132223	1.423993
H	0.087962	-1.303311	2.122009
H	0.479547	-5.079375	-1.069299
Te	0.248747	-3.809647	3.975871
H	2.069571	-4.348316	3.881422
C	3.554712	-2.810199	0.720274
C	3.419599	-1.640543	-0.023228
C	3.596902	-2.747681	2.115888
H	3.389020	-1.694842	-1.107234
C	3.298935	-0.411315	0.620347
C	3.476093	-1.512987	2.761694
H	3.186482	0.498249	0.038128
H	3.508250	-1.462640	3.845527
C	3.316860	-0.350024	2.012234
H	3.219497	0.605741	2.518022
H	3.615114	-3.770061	0.2171800
Te	3.849393	-4.512550	3.240796

MeXH/MeX$^{\bullet}$

MeOH E= -115.6547578

C	-0.046785	0.658091	0.000000
H	-1.091646	0.973590	0.000000
H	0.437523	1.077763	0.891937

H	0.437523	1.077763	-0.891937
O	-0.046785	-0.753213	0.000000
H	0.871595	-1.051960	0.000000

MeO$^{\bullet}$ E= -114.988968764

S^2= 0.7500 S^2_err%= 0%

C	-0.009738	-0.577810	0.000000
H	1.056999	-0.867510	0.000000
H	-0.460335	-1.006509	0.905992
H	-0.460335	-1.006509	-0.905992
O	-0.009738	0.793424	0.000000

TS(O) E= -230.635760987

S^2= 0.7500 S^2_err%= 0%

Nimag= -1809.67

O	1.332618	-0.130554	0.490813
H	2.006671	-0.077235	1.433628
O	2.820453	-0.518371	2.133057
C	2.132974	-0.394625	-0.621739
H	2.947898	0.328781	-0.738231
H	2.543247	-1.414321	-0.599874
H	1.470766	-0.324514	-1.494450
C	2.464528	-1.824683	2.472053
H	1.463109	-1.889868	2.912409
H	2.536189	-2.506190	1.612401
H	3.200238	-2.155100	3.216786

MeSH E= -438.627490861

C	-0.088590	0.791309	0.000000
H	-1.116860	1.155337	0.000000
H	0.412880	1.161787	0.894617
H	0.412880	1.161787	-0.894617
S	-0.190430	-1.027632	0.000000
H	1.131521	-1.260488	0.000000

MeS$^{\bullet}$ E= -437.990994106

S^2= 0.7500 S^2_err%= 0%

C	0.005248	-0.669131	0.000890
H	1.060614	-0.966977	-0.000885
H	-0.460498	-1.081283	0.897536
H	-0.459554	-1.078794	-0.897642
S	-0.028810	1.131284	0.000101

TS(S) E= -876.611571833

S^2= 0.7500 S^2_err%= 0%

Nimag= -1010.29

S	0.996064	0.260305	0.393038
H	2.048774	0.153697	1.561363
S	3.193735	-0.291370	2.548123
C	2.163244	-0.257408	-0.894243
H	3.009522	0.427352	-0.959183
H	2.515758	-1.277012	-0.728550
H	1.615072	-0.229349	-1.840050
C	2.610368	-2.003459	2.676076
H	1.595876	-2.046626	3.073906
H	2.659846	-2.516492	1.713964
H	3.283312	-2.509324	3.374022

MeSeH E= -2439.89234317

C	-0.105283	0.840314	0.000000
H	-1.129091	1.214059	0.000000
H	0.404604	1.182584	0.898338
H	0.404604	1.182584	-0.898338
Se	-0.238810	-1.106142	0.000000
H	1.225377	-1.331300	0.000000

MeSe$^{\bullet}$ E= -2439,26806523

S^2= 0.7500 S^2_err%= 0%

C	0.010284	-0.706465	0.000231
H	1.066025	-0.990439	-0.000435
H	-0.462651	-1.100207	0.898670
H	-0.462372	-1.098749	-0.899074
Se	-0.034286	1.230959	0.000608

TS(Se) E= -4879.16532530

S^2= 0.7501 S^2_err%= 0.01%

Nimag= -1023.96

Se	0.974302	0.353684	0.666800
H	2.307584	0.296509	1.729782
Se	3.658034	-0.568290	2.312161
C	2.058744	-0.110657	-0.884884
H	2.407963	0.792445	-1.381306
H	2.903098	-0.704588	-0.531910
H	1.441929	-0.704289	-1.559402
C	2.622237	-2.192297	2.611074
H	1.795082	-2.192701	1.899723
H	3.274938	-3.044868	2.423986
H	2.247661	-2.214632	3.632442

MeTeH E= -308.409327189

C	-0.128857	0.906861	0.000000
H	-1.147186	1.295878	0.000000
H	0.389083	1.240792	0.896427
H	0.389083	1.240792	-0.896427
Te	-0.293031	-1.233612	0.000000
H	1.352310	-1.468612	0.000000

MeTe$^{\bullet}$ E= -307.80855726

S^2= 0.7500 S^2_err%= 0%

C	0.016463	-0.749773	-0.000069
H	1.069752	-1.036805	-0.000255
H	-0.462649	-1.137567	0.896857
H	-0.462662	-1.136559	-0.897426
Te	-0.043904	1.395803	0.000894

TS(Te) E= -616.215655828

S^2= 0.7504 S^2_err%= 0.05%

Nimag= -897.12

Te	1.182875	0.971948	0.743148
H	2.592720	0.388990	1.868233
Te	3.883811	-0.777566	2.619277
C	2.127089	-0.030910	-0.903694
H	1.537263	0.187539	-1.794859
H	3.138724	0.350358	-1.027777
H	2.139912	-1.105014	-0.726592
C	2.442493	-2.366856	2.539508

H	2.180779	-2.578366	1.504117
H	2.906458	-3.246133	2.988283
H	1.559448	-2.083670	3.108823
